# Therapeutic potential of Akkermansia muciniphila in non-alcoholic fatty liver disease: a systematic review

**DOI:** 10.1186/s12876-025-04436-3

**Published:** 2025-11-19

**Authors:** Parastoo Asghari, Maryam Ahmadi-Khorram, Alireza Hatami, Saeedeh Talebi, Asma Afshari

**Affiliations:** 1 Department of Nutrition, Faculty of Medicine, Mashhad University of Medical Sciences, Mashhad, 91779-48564 Iran; 2 Student Research Committee, Mashhad University of Medical Sciences, Mashhad, Iran

**Keywords:** *Akkermansia muciniphila*, Microbiota, Non-alcoholic fatty liver disease

## Abstract

**Background:**

Non-alcoholic fatty liver disease (NAFLD) affects nearly one-third of the adult population worldwide, and currently, there are no approved pharmacological therapies. Akkermansia muciniphila, a bacterium found in the gut, has been identified as a promising therapeutic candidate due to its influence on the gut-liver axis.

**Objective:**

This systematic review aims to evaluate the efficacy of A. muciniphila in preclinical mouse models of NAFLD, focusing on its effects on body weight, glucolipid metabolism, liver function, gut barrier integrity, gut microbiota composition, inflammation, and immune response.

**Methods:**

Following PRISMA 2020 guidelines, a comprehensive search was conducted in PubMed, Scopus, Web of Science, and Google Scholar until September 30, 2025, for studies investigating A. muciniphila interventions in NAFLD mouse models. Inclusion criteria comprised mouse models of NAFLD, MAFLD, or NASH that involved A. muciniphila administration alongside a control group. Data were extracted concerning study characteristics, intervention details, and outcomes. The quality assessment of the studies was performed using the SYRCLE’s Risk of Bias tool.

**Results:**

Thirteen studies were included, predominantly employing C57BL/6 mice and high-fat diets. Results indicated that *A. muciniphila* reduced body weight, hepatic steatosis, and serum lipid levels, while improving insulin sensitivity and decreasing liver enzyme levels (ALT, AST). It also enhanced gut barrier function by upregulating tight junction protein expression and reducing lipopolysaccharide (LPS) translocation. Furthermore, anti-inflammatory effects were evidenced by decreased levels of TNF-α, IL-6, and MCP-1, alongside immunomodulation through the balance of Th17 and Treg cells.

**Conclusion:**

A. muciniphila exhibits potential in the management of preclinical NAFLD by improving metabolic, hepatic, and gut-related parameters. However, the absence of clinical trials limits the translatability of these findings. Future clinical investigations are imperative to establish efficacy, optimize dosing, and evaluate long-term safety.

**Trial registration:**

This systematic review has been documented with PROSPERO under the identifier: CRD42024610627.

**Graphical abstract:**

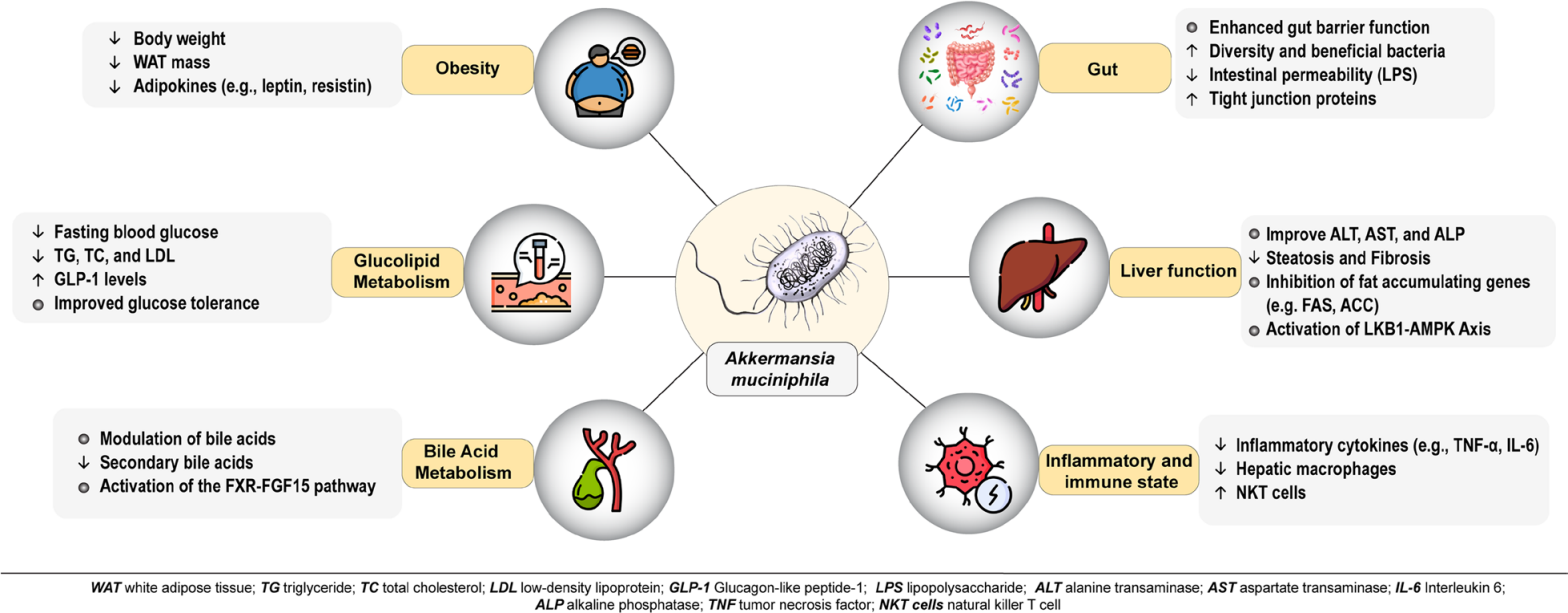

## Introduction

Non-alcoholic fatty liver disease (NAFLD) is the most prevalent chronic liver disorder worldwide, affecting approximately 32% of the global adult population, with a higher prevalence in males (40%) than females (26%) [1, 2]. Its incidence is anticipated to escalate in the forthcoming decade, as a result of the growing rates of obesity, the rise in type 2 diabetes, and an aging global population [3]. NAFLD encompasses a spectrum of conditions, ranging from simple hepatic steatosis to non-alcoholic steatohepatitis (NASH), characterized by fat accumulation, inflammation, and progressive fibrosis, which can lead to cirrhosis and hepatocellular carcinoma [4]. As the second leading cause of liver transplantation globally, NAFLD poses a significant public health challenge [5, 6].

The pathogenesis of NAFLD is complex, involving intricate interactions among metabolic, genetic, and environmental factors [7]. The “multiple-hit” hypothesis has largely replaced the earlier “two-hit” model, offering a more comprehensive framework for understanding the development of NAFLD. This hypothesis highlights the roles of insulin resistance, lipotoxicity, innate immune activation, and gut microbiota dysbiosis, all of which are influenced by genetic predisposition and lifestyle factors [8]. Despite advances in understanding these mechanisms, no FDA-approved pharmacological treatments for NAFLD currently exist, underscoring the urgent need for novel therapeutic strategies [9, 10]. Considering the role of dysbiosis within the multiple-hit hypothesis, the modulation of gut microbiota has emerged as a potentially effective therapeutic approach.

The gut–liver axis plays an essential role in NAFLD progression through the gut microbiota and their metabolites. Unhealthy lifestyles and disease complications lead to dysbiosis, identified by microbial imbalance, disrupted metabolites, and impaired barrier function, accelerating NAFLD development [11].

Among gut microbes, Akkermansia muciniphila, a Gram-negative, mucin-degrading bacterium from the Verrucomicrobia phylum, has garnered significant attention as a “next-generation probiotic” [12, 13]. A. muciniphila promotes gut barrier integrity, produces short-chain fatty acids (SCFAs), and reduces endotoxemia by limiting LPS translocation [13]. This bacterium, identified in 2004, is abundant in healthy individuals but reduced in both animals and humans with NAFLD [12, 14]. Studies have linked higher *A. muciniphila* abundance with reduced levels of liver enzymes (AST, ALT), total cholesterol, and triglycerides, suggesting its therapeutic potential in NAFLD [15]. A recent study found that reduced *Akkermansia* levels in HIV patients with metabolic disorders may predict NAFLD progression over 48 weeks of antiretroviral therapy [16]. However, the presence of confounding variables, such as diet and lifestyle factors, constrains the validity of the direct effects of *A. muciniphila* in these studies.

In light of the absence of clinical trials, this systematic review evaluates the consistency of findings across recent preclinical studies concerning the efficacy of *A. muciniphila* in mitigating NAFLD, examining its effects on metabolic, immune-inflammatory, and gut-related outcomes.

## Materials and methods

Complying with the Preferred Reporting Items for Systematic Reviews and Meta-Analyses statements 2020 [17], this systematic review was implemented and reported throughout all phases. This systematic review has been documented with PROSPERO under the identifier: CRD42024610627 (Registration Date: 16/11/2024).

### Search strategy

Relevant studies were identified through an unrestricted search of the PubMed, Scopus, Web of Science, and Google Scholar databases up to 30 September 2025. The references from the selected papers were carefully reviewed to identify any additional relevant studies. The search terms included: “*Akkermansia muciniphila*,” “non-alcoholic fatty liver disease,” “nonalcoholic steatohepatitis,” “NAFLD,” “NASH,” “steatohepatitis,” and “fatty liver.”

### Eligibility criteria

The inclusion criteria comprised: 1) mouse models of NAFLD, MAFLD, and NASH or obesity associated with these conditions; 2) intervention studies employing live or pasteurized *A. muciniphila* and its derived components; and (3) presence of a control group.

The exclusion criteria included: 1) studies lacking *A. muciniphila* administration; 2) studies utilizing other animal models; 3) mouse models of diseases or obesity unrelated to NAFLD, MAFLD, and NASH; 4) theses; 5) articles without full-text availability (abstracts); and 6) publications in languages other than English.

Studies involving additional simultaneous interventions with *A. muciniphila* were not excluded; only data pertaining to the *A. muciniphila* intervention group were reported.

Two independent investigators, P.A. and M.A., conducted the study selection process, strictly adhering to the inclusion criteria and excluding any studies that failed to meet these standards. They also performed data extraction and quality assessments. Any disagreements encountered at any stage were resolved through consensus.

### Data extraction

The author, year of publication, details of the intervention and control groups, the length and dosage of A. muciniphila administration, and the key results of NAFLD management were extracted from each study.

### Risk of bias assessment

The methodological quality of the studies included was assessed utilizing the SYRCLE risk of bias instrument, derived from the Cochrane risk of bias framework [18]. This instrument evaluates critical domains such as reporting bias, detection bias, performance bias, selection bias, and other pertinent biases. The maximum attainable score for each study was 10. Throughout the quality assessment procedure, any disagreements were deliberated upon and resolved in consultation with the respective author.

## Results

### Literature selection

**Fig. 1:**
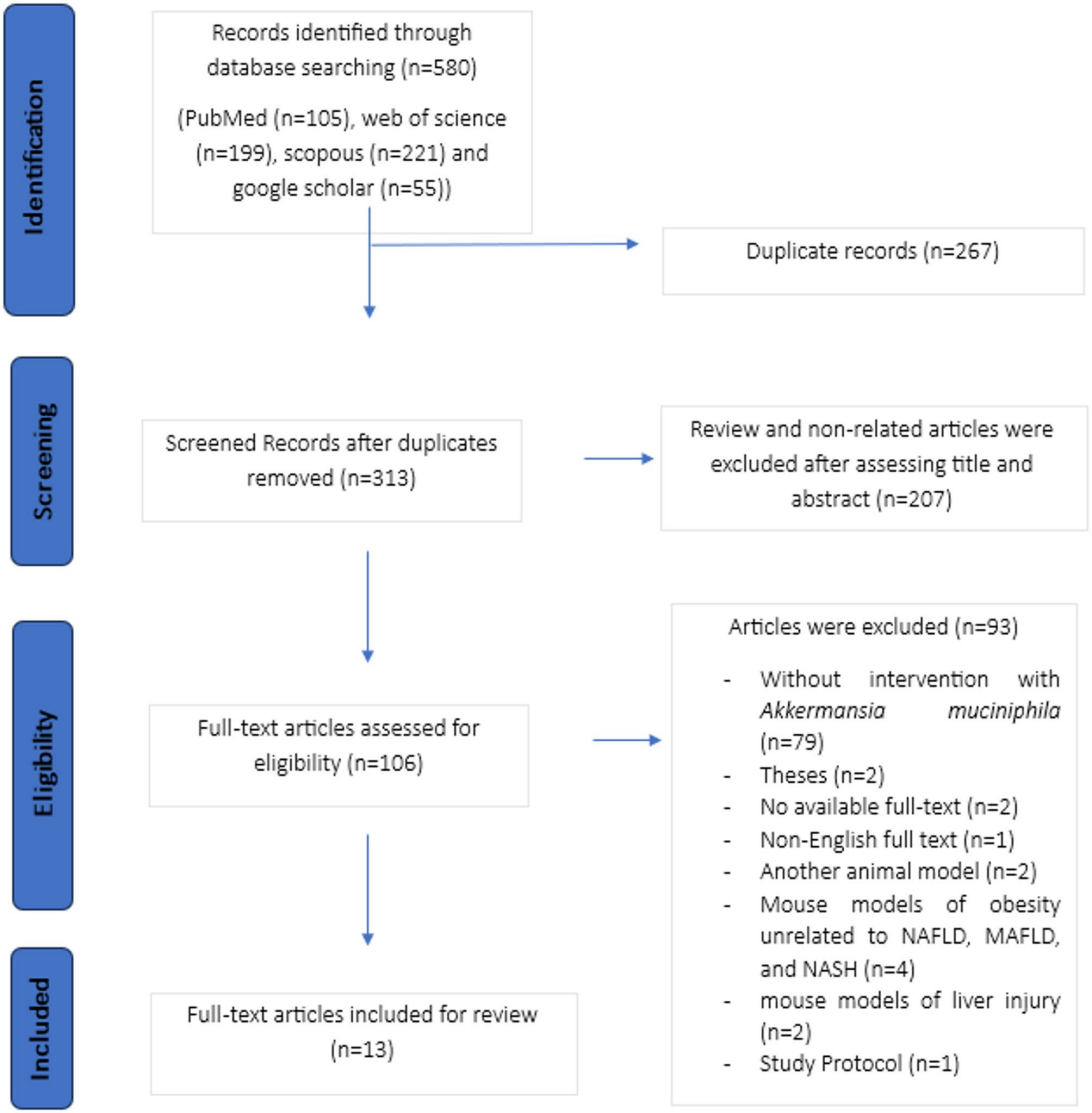
A PRISMA flowchart outlining the study selection process

A total of 580 articles were retrieved from the databases using the predetermined search strategy. After eliminating 267 duplicate studies, 313 articles were initially screened based on their titles and abstracts. Out of these, 207 articles were subsequently excluded. The remaining 106 papers were then evaluated in accordance with the study’s inclusion and exclusion criteria. Ultimately, 13 studies [19–31] were selected for review (Fig. 1). The observation that all the included studies were published between 2021 and 2025 indicates a growing interest in examining the potential of *A. muciniphila* for the treatment of NAFLD in recent years.

### Characteristics of included studies

All studies were published in English. The studies mainly used C57BL/6 (C57BL/6J) mice, with one study employing Wistar rats [26] Moreover, another using Ldlr−/−.Leiden mice [24]Furthermore one study [31]used ICR mice to establish the germ-free (GF) mouse model. Male animals were used in all but one study, which included both male and female mice [28]. The initial weight of the animals was reported as 18–20 g in one study [27], 20 ± 2 g in two studies [20, 29], and not specified in the remaining ten studies. Animal age was documented in twelve studies, ranging from 4 days [25] to 15–17 weeks [24]. Regarding the type of *A. muciniphila*, eight studies used the ATCC BAA-835 strain, one study used CIP-107961T [26], one study used both AM02 (feces-isolated) and AM06 (breast milk-isolated) [25], three studies did not specify the strain used. Live bacteria were administered in eleven studies, while two studies used pasteurized bacteria [20, 24]. The duration of *A. muciniphila* administration ranged from 2 to 28 weeks, with the highest dosage reported as 5 × 10^9^ CFU/mL [30]. The experimental models were mainly built using a high-fat diet (HFD) or its variations, including high-fat high-cholesterol (HFC/HFHC), high-fat high-fructose (HFHFD), or high-fat high-carbohydrate (HFHCD) diets in ten studies. Two studies used a methionine- and choline-deficient (MCD) diet [19], one employed the STAM model (streptozotocin injection followed by HFD) [25], and one used carbon tetrachloride (CCl₄) injections to induce liver cirrhosis [28]. The route of administration was mainly oral gavage, with one study using diet supplementation [24]. Administration frequency varied, including daily, twice daily, every other day, three times weekly, or twice weekly, with two studies not reporting the frequency [26, 29]. Table 1 summarizes the detailed characteristics of the included studies.

**Table 1:** Basic characteristics of the included studies

First author (year)	Species (sex, age, weight)	Disease Model construction (Disease, method, induction time)	A. Muciniphila administration (type, form, dosage, route, frequency, duration)
Hu et al. (2025) [29]	C57BL/6 mice (Male, 5-weeks-old, 20 ± 2 g)	(MAFLD, 60% kcal fat diet (HFD) for 12 weeks or choline-deficient high-fat diet (CD-HFD) for 6 weeks)	(BAA-835, ATCC, live, 0.2 mL PBS (1 × 10^9^ CFU/mL), oral gavage, frequency NR, 4 weeks)
Wu et al. (2025) [30]	C57BL/6 mice (Male, 14 days old for DEN injection, 8 weeks old for HFHC, weight NR)	(MAFLD-related HCC, DEN + HFD (32 weeks), HFHC diet (32 weeks), orthotopic MAFLD-HCC (normal diet, 24 weeks; MCD diet, 2 weeks; HFHC diet, 24 weeks)	(Type NR, Live, 200 µL PBS solution (5 × 10^9^ CFU/mL), oral, gavage, daily, 8 weeks for (MAFLD-HCC HFHC))
Zhuge et al. (2025) [31]	SPF C57BL/6J mice (Male, 6 weeks old, NR)	(MAFLD, HFHFD (60 kcal% fat) for 0, 4, 8, 12, or 16 weeks)	(ATCC BAA-835, live, normal saline 10^9^ CFU/mouse, oral gavage, twice weekly for the MAFLD model, 20 weeks)
Oguri et al. (2024) [28]	SPF wild-type C57BL/6J mice (male and female, 4 weeks old at start, 8–14 weeks during CCl4, 14–18 weeks during Akk, weight NR)	(LC, CCl4/olive oil intraperitoneal injection, 6 weeks)	(ATCC BAA-835, live, 200 µL PBS with 20% glycerol (1 × 10^10^ CFU/mL) solution, oral gavage, daily, 4 weeks)
Wu et al. (2024) [19]	C57BL/6 J mice (male, 6-week-old, weight NR)	(NAFLD/NASH, MCD diet, 8 weeks)	(BAA-835, live, 200 µL normal saline (2 × 10^8^ CFU), oral gavage, daily, 8 weeks)
Qu et al. (2023) [20]	SPF C57BL/6 mice (male, aged 5–6 weeks, 20 ± 2 g)	(NAFLD, HFD (60% kcal from fat, 10 weeks)	(Type NR, Pasteurized, PBS solution (1.5 × 10^9^ CFU/200 µL), oral gavage, frequency NR, 10 weeks)
Nian et al. (2023) [21]	SPF C57BL/6 mice (male, age NR, weight NR)	(NAFLD, HFD (40% kcal from fat, 20 weeks)	(Type NR, live, 0.2 ml PBS suspension (10^9^ CFU/mL), oral gavage, 3 times/week, 8 weeks)
Wu et al. (2023) [22]	SPF C57BL/6 mice (male, 6-week-old, weight NR)	(MAFLD, HFD (60% fat energy), 21 weeks)	(ATCC BAA-835, live, 0.2 ml PBS suspension (~ 1.5 × 10^10^ CFU/mL), oral gavage, daily, 21 weeks)
Han et al. (2023) [23]	Wild-type C57BL/6 mice (male, 6-week-old, weight NR)	(NASH, HFD or HFHCD (60% fat energy, 20 weeks)	(ATCC BAA − 835, live, 10^9^ CFU in 200 µL sterile anaerobic PBS with 2.5% glycerol, oral gavage, daily, 20 weeks)
Morrison et al. (2022) [24]	Ldlr−/−. Leiden mice (male, aged 15–17 weeks, weight NR)	(Obesity-associated NAFLD/NASH, HFD (45 kcal% fat from lard), 28 weeks)	(ATCC BAA-835, pasteurized, lyophilized at 7.14 × 10^10^ heat-inactivated CFU/kg diet, delivering 2 × 10^8^ heat-inactivated CFU/mouse/day, based on 2.8 g daily food intake diet supplementation, daily, 28 weeks)
Li et al. (2022) [25]	Wild-type SPF C57BL/6J, CD1d-knockout, CXCR6-knockout mice (Male, 4 days old at STZ injection, weight NR)	(NASH-to-HCC, STZ (200 µg) at 4 days + HFD (60% fat energy) at 4 weeks, 6, and 16 weeks)	(AM06 and AM02, live, PBS solution (1.0 × 10^9^ CFU), oral gavage, twice daily, 4 weeks (baseline), 10 weeks (STAM/other models)
Juárez-Fernández et al. (2021) [26]	Wistar rats (male, 21-day-old, weight NR)	(juvenile model of obesity and NAFLD, HFD (60% fat energy), 6 weeks)	(CIP-107961T, live, 2 × 10^8^ CFU in 200 µL cysteine-supplemented (10%) skim milk, oral gavage, frequency NR*, 3 weeks)
Rao et al. (2021) [27]	C57BL/6 mice (male, aged 7–8 weeks, 18–20 g)	(Obesity and MAFLD, HFC diet (60% high fat and 1.2% cholesterol), 10 weeks)	(ATCC BAA-835, live, 200 µL PBS suspension at 1 × 10^8^ CFU/ml), oral gavage, every other day, 6 weeks)

*AM02* A.muciniphila isolated from feces, *AM06* A.muciniphila isolated from breast milk, *Amuc_1100* Outer membrane protein of A.muciniphila, *CCl4* Carbon tetrachloride, *CD-HFD* Choline-deficient high-fat diet, *CFU* Colony-forming unit, *DEN* Diethylnitrosamine, *HCC* Hepatocellular carcinoma, *HFC/HFHC* High-fat and high-cholesterol diet, *HFD* High-fat diet, *HFHCD* High-fat high-carbohydrate diet, *HFHFD* High-fat high-fructose diet, *LC* Liver cirrhosis, *MAFLD* Metabolic-associated fatty liver disease, *MCD* Methionine- and choline-deficient diet, *NAFLD* Non-alcoholic fatty liver disease, *NASH* Non-alcoholic steatohepatitis, *NR* Not Reported, *SPF* Specific pathogen-free, *STZ* Streptozotocin

### Quality of included studies

Each study was assigned a score ranging from three to five, indicating a low to moderate overall quality, thereby implying that the results should be interpreted with caution. Regarding baseline characteristics, all studies demonstrated a low risk of bias, with the exception of two studies [25, 26]. Specifically, all included studies exhibited unclear risks regarding sequence generation, allocation concealment, randomization, blinding of researchers, and random assessment. Regarding the blinding of outcome assessors, three studies [25, 26, 29] Reported a low risk, whereas the remaining studies were inconclusive. Concerning incomplete outcome data, ten studies demonstrated a low risk, whereas two studies [24, 25] exhibit high risk; one study was characterized by an unclear risk of bias [23]. All studies demonstrated a low risk for selective outcome reporting. Table 2 delineates the results of the study quality assessment, as evaluated by SYRCLE’s Risk of Bias tool.

**Table 2:** Assessment of included articles according to SYRCLE's criteria

Study	Sequence generation	Baseline characteristics	Allocation concealment	Random housing	Blinding researcher	Random assessment	Blinding outcome assessors	Incomplete outcome data	Selective outcome reporting	Others	Total
Hu et al. (2025)	U	L	U	U	U	U	L	L	L	L	5
Wu et al. (2025)	U	L	U	U	U	U	U	L	L	L	4
Zhuge et al. (2025)	U	L	U	U	U	U	U	L	L	L	4
Oguri et al. (2024)	U	L	U	U	U	U	U	L	L	L	4
Wu et al. (2024) [19]	U	L	U	U	U	U	U	L	L	L	4
Qu et al. (2023) [20]	U	L	U	U	U	U	U	L	L	U	3
Nian et al. (2023) [21]	U	L	U	U	U	U	U	L	L	L	4
Wu et al. (2023) [22]	U	L	U	U	U	U	U	L	L	U	3
Han et al. (2023) [23]	U	L	U	U	U	U	U	U	L	L	3
Morrison et al. (2022) [24]	U	L	U	U	U	U	U	H	L	L	3
Li et al. (2022) [25]	U	U	U	U	U	U	L	H	L	L	3
Juárez-Fernández et al. (2021) [26]	U	U	U	U	U	U	L	L	L	L	4
Rao et al. (2021) [27]	U	L	U	U	U	U	U	L	L	L	4

*L* Low risk of bias, *H* High risk of bias, *U* Unclear risk of bias

### Main results of selected studies

The findings of the interventional studies, which are compiled in Table 3, serve as the basis for the results presented below.

#### Effect of *Akkermansia muciniphila* on body weight, food intake, adipocytes and adipokines

Obesity and the increase in white adipose tissue (WAT) mass are significant risk factors for NAFLD. They contribute to disease progression through the dysregulation of adipokine secretion, including a reduction in adiponectin levels, which consequently results in insulin resistance and ectopic fat accumulation [32, 33]. Adipokines regulate energy metabolism, inflammation, and fibrosis [34].

Eight studies have documented the anti-obesity effects of *A. muciniphila* [20–22, 26–29, 31]. However, their findings were influenced by various factors, including bacterial strain and preparation, viability, dosage, host model, and dietary conditions. For instance, Hu et al. [29], Zhuge et al. [31], Wu et al.(2023) [22], and Oguri et al. [28] reported that oral administration of live *A. muciniphila* (ATCC BAA-835) reduced body weight gain in C57BL/6 mice fed a high-fat diet. Specifically, Hu et al. [29] administered 0.2 mL PBS (1 × 10^9^ CFU/mL) for 4 weeks, Zhuge et al. utilized 10⁹ CFU/mouse biweekly over 20 weeks, Wu et al.(2023) [22] used 0.2 ml PBS suspension (~ 1.5 × 10^10^ CFU/mL) daily during 21 weeks, and Oguri et al. [28] administered 200 µL of PBS containing 20% glycerol (1 × 10¹⁰ CFU/mL) daily for a duration of 4 weeks to mice with CCl4-induced liver cirrhosis, resulting in a trend towards reduced body weight gain compared to the control group. Meanwhile, Juárez-Fernández et al. [26] reported that three weeks of oral treatment with 2 × 10⁸ CFU of live *A. muciniphila* (CIP-107961T) in 200 µL of cysteine-supplemented (10%) skim milk led to reductions in body weight and white adipose tissue (WAT) mass in Wistar rats exhibiting early obesity and NAFLD. Interestingly, Qu et al. [20] demonstrated that 1.5 × 10⁹ CFU in 200 µL of *A. muciniphila* and its outer membrane protein Amuc_1100, administered for 10 weeks, decreased body weight in C57BL/6 mice on a high-fat diet. It is noteworthy that pasteurized *A. muciniphila* also shows efficacy, suggesting that non-viable bacterial components may retain anti-obesity properties. Nian et al. [21] and Rao et al. [27] reported a 20.8% decrease after every-other-day gavage with 200 µL of a 1 × 10⁸ CFU/mL PBS suspension (ATCC BAA-835), whereas Nian et al. [21] found that three doses per week of 0.2 mL from a 10⁹ CFU/mL suspension over eight weeks reduced body weight compared to controls, although the bacterial strain was not specified. Conversely, three studies [19, 24, 25] reported no significant effects on body weight, potentially due to differences in bacterial preparation, dosage, administration route, mouse strain, or dietary interventions. For instance, Morrison et al. [24] observed that heat-inactivated *A. muciniphila* (ATCC BAA-835) administered daily at 2 × 10⁸ CFU per mouse for 28 weeks did not influence weight gain in Ldlr−/− Leiden mice subjected to a high-fat diet, nor did it affect WAT depots, fat mass, lean body mass, or food intake, indicating the importance of bacterial viability for efficacy. Similarly, Wu et al. [19](2024) found that eight weeks of daily oral gavage of live *A. muciniphila* (BAA-835, 2 × 10⁸ CFU in 200 µL saline) had no impact on body weight in mice fed a MCD diet. Li et al. [25] reported that administering live *A. muciniphila* (AM06) at 1.0 × 10⁹ CFU twice daily for ten weeks did not alter the reduced body weight in STZ-injected and HFD-fed C57BL/6J mice, suggesting that the benefits of AM06 may not depend on weight modulation. In contrast, Han et al. [23] reported that oral gavage of live *A. muciniphila* (ATCC BAA-835, 10⁹ CFU in 200 µL PBS with 2.5% glycerol) reduced body weight in HFD-fed C57BL/6 mice by week 16 despite unchanged food intake, although no effects were observed in mice fed a normal diet after 20 weeks. Overall, despite variations among studies, *A. muciniphila* appears more effective in reducing weight at higher doses and within high-fat diet contexts.

Regarding adipose tissue, Wu et al.(2023) [22] found that daily oral administration of 0.2 mL PBS suspension (~ 1.5 × 10¹⁰ CFU/mL) of live *A. muciniphila* (ATCC BAA-835) for 21 weeks resulted in smaller adipocytes and reductions in subcutaneous, mesenteric, and epididymal WAT, along with decreased serum levels of insulin, leptin, and resistin in HFD-fed mice, without affecting adiponectin levels. Conversely, Morrison et al. [24] reported that dietary supplementation with 2 × 10⁸ CFU of heat-inactivated *A. muciniphila* (ATCC BAA-835) suppressed mesenteric WAT hypertrophy but did not influence overall WAT mass or inflammation in high-fat diet Ldlr−/− Leiden mice. This suggests that higher dosages of live *A. muciniphila* may have superior effects on different adipose tissue depots. Supporting this, Juárez-Fernández et al. [26] observed a decrease in WAT in rats after three weeks of oral gavage with 2 × 10⁸ CFU of live *A. muciniphila* (CIP107961T).

#### Effect of *Akkermansia muciniphila* on glucolipid metabolism

NAFLD denotes the liver’s response to dysregulated lipid and glucose metabolism, frequently associated with obesity, dyslipidemia, and insulin resistance [35]. Insulin resistance contributes to hepatic lipid accumulation and facilitates the progression to non-alcoholic steatohepatitis (NASH) and, potentially, hepatocellular carcinoma [36].

##### Glucose metabolism

Numerous studies have explored the impact of live *A. muciniphila* supplementation on glucose metabolism in mice subjected to HFD, indicating predominantly beneficial yet inconsistent results depending on strain, dose, and treatment duration. For instance, Zhuge et al. [31] demonstrated that oral gavage of 10⁹ CFU/mouse of live *A. muciniphila* (ATCC BAA-835) twice weekly for 20 weeks normalized the impaired insulin sensitivity induced by high-fat high-cholesterol diet (HFHFD) in C57BL/6J mice, as evidenced by enhanced IGTT results and restored fasting insulin levels. Similarly, Nian et al. [21] reported reductions in fasting blood glucose and improved insulin sensitivity following eight weeks of thrice-weekly oral gavage with 10⁹ CFU/mL live bacteria. Wu et al.(2023) [22] observed improved glucose tolerance in HFD-fed C57BL/6 mice after daily administration of a higher dose (~ 1.5 × 10¹⁰ CFU/mL) for 21 weeks. Li et al. [25], using live strain AM06 at a dose of 1.0 × 10⁹ CFU twice daily, noted lower fasting glucose and increased GLP-1 levels after ten weeks, although insulin levels remained unchanged in STZ-injected and HFD-fed mice. Han et al. [23] found that oral gavage of live *A. muciniphila* (ATCC BAA − 835, 10⁹ CFU in 200 µL of PBS) improved glucose response by week 11 in HFD-fed C57BL/6 mice, reducing blood glucose levels at 30 min and the area under the curve in glucose tolerance tests (GTT). By week 18, serum glucose levels normalized, but no effects were observed in mice fed a normal diet after 20 weeks, suggesting a more pronounced effect in the context of diet-associated metabolic disorders. Suggesting a more pronounced effect in the context of diet-associated metabolic disorders. Conversely, Morrison et al. [24] reported no changes in blood glucose or insulin levels after 28 weeks of daily dietary supplementation with 2 × 10⁸ CFU/mouse heat-inactivated *A. muciniphila* (ATCC BAA-835) in HFD-fed Ldlr−/−. Leiden mice, emphasizing the significance of bacterial viability in relation to metabolic effectiveness.

##### **Lipid metabolism**

Multiple studies have explored the effects of Akkermansia muciniphila (AKK) supplementation on serum lipid profiles, with most indicating reductions in triglyceride (TG) and total cholesterol (TC) levels. For instance, Hu et al. [29] and Rao et al. [27] reported that oral administration of live *A. muciniphila* (ATCC BAA-835) showed lipid-lowering effects in C57BL/6 MAFLD mice fed high-fat (HF) diets. Specifically, Hu et al. [29] reported reduced TG levels after administering 0.2 mL PBS containing 10⁹ CFU/mL for four weeks, and Rao et al. [27] noted decreased hepatic and plasma cholesterol following administration of 200 µL PBS suspension containing 10⁸ CFU/mL every other day for six weeks. Similarly, Nian et al. [21] observed decreased serum TG and TC levels following eight weeks of treatment with live *A. muciniphila* (1 × 10⁹ CFU/mL, 0.2 mL PBS) administered three times weekly. In line with these findings, Wu et al. (2025) [30] demonstrated that daily oral gavage of live *A. muciniphila* (200 µL at 5 × 10⁹ CFU/mL) for eight weeks reduced serum TC and TG levels in MAFLD-HCC models, irrespective of whether mice were fed a methionine–choline-deficient or high-fat high-cholesterol diet.

Pasteurized *A. muciniphila* and its bacterial constituents exhibited lipid-lowering properties. Que et al. [20] demonstrated that pasteurized *A. muciniphila* (1.5 × 10⁹ CFU/200 µL) and its outer membrane protein Amuc_1100 for ten weeks significantly reduced serum TC, TG, and LDL-C levels in HFD-fed C57BL/6 mice. Similarly, Wu et al.(2024) [19] also observed that administration of live *A. muciniphila* (BAA-835; 2 × 10⁸ CFU in 200 µL saline) via oral gavage for eight weeks in C57BL/6J mice subjected to an MCD diet resulted in lower TG and LDL-C levels, although HDL levels remained unaffected, indicating selective modulation of lipid metabolism.

Nevertheless, results across studies are not uniformly consistent. Zhuge et al. [31] discovered that A. muciniphila improved TC levels but did not significantly influence serum TG levels. Han et al. [23] that oral gavage of live *A. muciniphila* (ATCC BAA − 835, 10⁹ CFU/mouse) did not initially reduce TC levels in HFD-fed C57BL/6 mice, although levels normalized by week 18. Moreover, Li et al. [25] reported no change in TG levels in STZ-injected and HFD-fed C57BL/6J mice following the oral administration of live A. muciniphila (AM06) at a dose of 1.0 × 10⁹ CFU twice daily for ten weeks, and after 28 weeks, Morrison et al. [24] found no effect of daily dietary supplementation with heat-inactivated A. muciniphila (ATCC BAA-835) (2 × 10⁸ CFU/mouse) on plasma lipids.

#### Effect of *Akkermansia muciniphila* on liver function, hepatic steatosis, hepatic fibrosis, and steatohepatitis

NAFLD progresses from impaired liver function and hepatic steatosis to, in more advanced cases, NASH accompanied by fibrosis, which may ultimately lead to cirrhosis. Key indicators comprise elevated liver enzymes (ALT, AST, ALP) and histological changes, such as lipid accumulation and inflammatory responses.

##### Liver function

*A. muciniphila* supplementation demonstrated hepatoprotective effects across multiple studies in a dose- and duration-dependent manner. Qu et al. [20] reported that both pasteurized A. muciniphila (1.5 × 10⁹ CFU/200 µL) and its outer membrane protein Amuc_1100, administered over a 10-week period, reduced serum ALT and AST levels in high-fat diet (HFD)-fed C57BL/6 mice, indicating attenuation of hepatocellular damage. Similarly, Han et al. [23], Wu et al.(2023) [22], and Rao et al. [27] demonstrated that oral gavage of live *A. muciniphila* (ATCC BAA-835) significantly improved liver enzyme profiles in mice fed high-fat and high-carbohydrate diets (HFD, HFC). Specifically, Han et al. [23] administered 200 µL PBS (10^9^ CFU) for 16 weeks, whereas Wu et al.(2023) [22] delivered 0.2 mL PBS suspension (~ 1.5 × 10^10^ CFU/mL) daily over 21 weeks, both resulting in notable decreases in serum ALT and AST. Correspondingly, Rao et al. [27] found that alternate-day gavage (1 × 10⁸ CFU/mL, 200 µL) for six weeks led to reductions in ALT and AST levels, as well as a decrease in ALP levels, with effects persisting after treatment cessation. Conversely, Wu et al.(2023) [22] observed that in mice fed a standard diet, continual administration of *A. muciniphila* for twenty weeks did not significantly alter AST and ALT levels, suggesting effects specific to metabolic impairment. Regarding the effects of live BAA-835 strain, Zhuge et al. [31] also discovered that twice-daily gavage of 10⁹ CFU for 20 weeks alleviated elevated ALT and AST levels induced by a high-fat and high-fructose diet, while Hu et al. [29] documented decreased ALT levels and mitigation of MAFLD progression following a 4-week treatment period (1 × 10⁹ CFU/mL, 0.2 mL). Oguri et al. [28] further observed enhanced ammonia metabolism, as evidenced by reduced blood NH₃ concentrations after 4 weeks of daily oral administration of live ATCC BAA-835 (1 × 10⁹ CFU, 200 µL) in mice with liver cirrhosis. In contrast, Wu et al.(2024) [19] reported no improvement in ALT levels among mice fed a methionine-choline-deficient (MCD) diet after eight weeks of daily oral introduction of live *A. muciniphila* (BAA-835, 200 µL, 2 × 10⁸ CFU), while Morrison et al. [24] noted no effect from the daily dietary administration of heat-inactivated *A. muciniphila* (ATCC BAA-835, 2 × 10⁸ CFU/mouse, 28 weeks). This indicates that the beneficial effects are dependent upon both bacterial viability and the delivery method. Regarding the AM06 strain, Li et al. [25] reported that increased ALT levels observed in NASH-HCC mice did not improve following administration of *A. muciniphila*; however, AST levels appeared to decrease post-treatment.

##### **Hepatic steatosis**,** fibrosis**,** and steatohepatitis**

*A. muciniphila* attenuated hepatic steatosis; however, the effects on fibrosis and steatohepatitis were less consistent. Han et al. [23] reported that oral gavage of live *A. muciniphila* (ATCC BAA − 835; 10⁹ CFU per mouse) in high-fat diet-fed C57BL/6 mice resulted in reductions in liver steatosis, NAS score, and inflammatory infiltration by week 20. A slight reduction in hepatic fibrosis was observed by week 26, indicating a time-dependent response. Li et al. [25] observed that the live *A. muciniphila* strain AM06 (10⁹ CFU administered twice daily for 10 weeks) diminished fatty liver degeneration, NAS, steatosis, ballooning scores, and inflammatory cell infiltration in NASH mice, although lobular inflammation persisted. In contrast, strain AM02 exhibited no effect, thereby emphasizing strain-specific efficacy. Similarly, Wu et al. [19] Wu et al. observed a reduction in lipid accumulation and hepatocyte ballooning in mice fed with MCD following eight weeks of daily gavage with 2 × 10⁸ CFU of the live strain BAA-835. while Zhuge et al. [31] confirmed improvements in NAS and hepatic lipid accumulation following a 20-week regimen of twice-daily oral gavage with 10⁹ CFU. Additionally, Oguri et al. [28] found that a four-week regimen of daily gavage with the live ATCC BAA-835 strain (10⁹ CFU/day) in a CCl₄-induced fibrosis model markedly diminished liver fibrosis. In contrast, Morrison et al. [24] Reported no reduction in steatosis, inflammation, or fibrosis following 28 weeks of daily dietary supplementation (heat-inactivated, ATCC BAA-835, 2 × 10⁸ CFU/mouse), implying that the bacterial delivery method or viability influences outcomes.

Across multiple studies, A. muciniphila supplementation consistently reduced hepatic lipid accumulation: Wu et al. [22] demonstrated that a daily administration of live A. muciniphila (ATCC BAA-835; approximately 1.5 × 10¹⁰ CFU/mL, over a period of 21 weeks) resulted in a reduction of liver triglycerides and liver volume. Similarly, Qu et al. [20] Reported a decrease in lipid droplet formation with pasteurized bacteria (1.5 × 10⁹ CFU/200 µL) or Amuc_1100 protein over a period of 10 weeks. Juárez-Fernández et al. [26] found that a lower daily dose of live strain (CIP-107961T; 2 × 10⁸ CFU, 3 weeks) reduced hepatic triglycerides, while Nian et al. [21] demonstrated that intermittent dosing (10⁹ CFU, administered three times weekly for a duration of 8 weeks) mitigated steatosis.

The oral administration of live *A. muciniphila* (ATCC BAA-835) exerts a significant hepatoprotective effect: Rao et al. [27] reported that alternate-day gavage of 200 µL suspension (1 × 10⁸ CFU/mL) for six weeks resulted in a reduction of hepatic diacylglycerol levels and maintained lower hepatic triglyceride concentrations even after the cessation of treatment in HFC-fed C57BL/6 mice. Similarly, Wu et al. [30]found the daily oral gavage of 200 µL PBS solution (5 × 10⁹ CFU/mL) for eight weeks significantly downregulated hepatic genes associated with cholesterol biosynthesis, consequently reducing steatosis in MAFLD-HCC mice subjected to MCD or HFHC diets. Consistently, Hu et al. [29] The study demonstrated that the daily administration of 0.2 mL PBS suspension (1 × 10⁹ CFU/mL) resulted in a reduction in hepatic lipid accumulation, inflammation, and liver injury, while simultaneously promoting liver regeneration in comparison to PBS-treated control subjects.

#### Effect of *Akkermansia muciniphila* on gut barrier function

Impaired gut barrier function, characterized by increased intestinal permeability, disrupted tight junctions, and bacterial translocation, exacerbates NAFLD by promoting hepatic inflammation via the gut-liver axis [37].

Eleven studies [19–24, 27–31] demonstrated that *A. muciniphila* enhances gut barrier function. Wu et al. [19] observed that oral gavage of live *A. muciniphila* (BAA-835, 2 × 10⁸ CFU) for eight weeks restored intestinal epithelial integrity, repaired villi damage, and increased the expression of tight junction proteins (Cldn-1, ZO-2) in mice fed with a methionine-choline-deficient (MCD) diet. Que et al. [20] reported that after administration of ten weeks of pasteurized *A. muciniphil*a (1.5 × 10⁹ CFU/200 µL) and its outer membrane protein Amuc_1100, there was an upregulation of ZO-1, Occludin, and Claudin-1 in the colon of high-fat diet (HFD)-fed mice, along with a reduction in serum LPS levels. Similarly, Nian et al. [21] demonstrated improved villus organization and lamina propria structure, reduced intestinal permeability, and increased expression of ZO-1 and Occludin following three weekly doses of live *A. muciniphila* (0.2 mL, 10⁹ CFU/mL) for eight weeks. Wu et al. [22] showed that daily gavage of 0.2 mL live *A. muciniphila* (~ 1.5 × 10¹⁰ CFU/mL) for 21 weeks led to a thicker mucus layer, upregulated occludin and Tjp1 expression, and decreased intestinal permeability, as evidenced by lower plasma FD4 and LBP levels. Han et al. [23] found that administration of live *A. muciniphila* (10⁹ CFU, 200 µL PBS) improved epithelial integrity, restoring tight junction proteins (Ocln, Cldn23, Muc2, ZO-1, Cldn1, Tjp3) and mucin production in mice fed an HFD by week 14. Conversely, Morrison et al. [24] reported that heat-inactivated *A. muciniphila* (2 × 10⁸ CFU/mouse) administered daily for 28 weeks gradually enhanced gut barrier function but did not prevent initial HFD-induced permeability; however, it reduced PRO-C4, a marker of collagen turnover associated with gut integrity, suggesting that live strains outperform non-viable strains in protecting the gut barrier. Additionally, Rao et al. [27] reported that alternate-day gavage of live *A. muciniphila* (1 × 10⁸ CFU/200 µL) for six weeks in high-fat, choline-deficient (HFC)-fed mice resulted in a thicker intestinal mucosa, higher E-cadherin expression, and shorter ileal villi, alongside a reduction in plasma LPS levels, indicating improved barrier integrity.

Oguri et al. [28] observed elevated levels of Cldn2, Cldn3, and Occludin following daily gavage of live *A. muciniphila* (ATCC BAA-835, 1 × 10⁹ CFU/200 µL PBS with 20% glycerol) for four weeks. Hu et al. [29] discovered that oral gavage of 0.2 mL live *A. muciniphila* (1 × 10⁹ CFU/mL) maintained gut barrier integrity by increasing mucosal thickness, enhancing E-cadherin expression, and decreasing serum lipopolysaccharide (LPS) levels. Wu et al. [30] reported that administration of live *A. muciniphila* (5 × 10⁹ CFU/200 µL) for eight weeks improved the expression of tight junction proteins (ZO-1, Occludin, Claudin-1/4) and reduced LPS flux in mice with metabolic dysfunction-associated fatty liver disease (MAFLD)-hepatocellular carcinoma (HCC) fed high-fat, high-cholesterol (HFHC) and methionine-choline-deficient (MCD) diets. Furthermore, Zhuge et al. [31] indicated that twice-weekly gavage of 10⁹ CFU/mouse of live *A. muciniphila* (ATCC BAA-835) for 20 weeks enhanced the thickness of the mucus layer and the expression of tight junction genes in mice fed a high-fat, high-fructose diet.

#### Effect of *Akkermansia muciniphila* on gut microbiota composition

Gut microbiota dysbiosis, characterized by diminished α-diversity, altered β-diversity, and fluctuations in bacterial abundance, contributes to the progression of NAFLD by disrupting the gut-liver axis, impairing gut barrier function, and modifying metabolite production (e.g., lipopolysaccharides [LPS], short-chain fatty acids [SCFAs], bile acids [BAs]) [38].

*A. muciniphila* modulates gut microbiota. Fernández et al. [26]. observed an increase in the abundance of the Verrucomicrobia phylum and the Akkermansia genus in rats fed a high-fat diet (HFD), which directly corresponded to enrichment following oral gavage of 2 × 10^8 CFU of live *A. muciniphila* (CIP-107961T) over a period of three weeks. Qu et al. [20], observed that a ten-week intervention with pasteurized *A. muciniphila* (1.5 × 10^9 CFU/200 µL) and its outer membrane protein Amuc_1100 partially reversed microbiota alterations induced by a high-fat diet (HFD), notably decreasing Erysipelotrichaceae and Atopobiaceae, while increasing Lachnospiraceae and Ruminococcaceae. Additionally, Amuc_1100 reduced Coriobacteriaceae_UCG-002 levels and promoted the growth of beneficial genera such as Blautia and Lachnoclostridium.

Wu et al. [19] reported that oral gavage of live A. muciniphila (BAA-835, 2 × 10^8 CFU) over an eight-week period resulted in an increase in Proteobacteria and β-diversity in mice fed with a methionine-choline deficient diet (MCD). The study observed enrichment in Tannerellaceae, Enterobacteriaceae, Gammaproteobacteria, and Faecalibaculum rodentium, although α-diversity remained low. Nian et al. [21] observed an increased abundance of *Faecalibaculum*,* Lactobacillales*, and *Adlercreutzia*, alongside a decrease in *Romboutsia*,* Lactobacillus*, and *Parabacteroides*, which may potentially contribute to the protection against NAFLD subsequent to three weekly administrations of live A. muciniphila (0.2 mL, 10⁹ CFU/mL) over a period of eight weeks. Rao et al. [27] reported that administering live *A. muciniphila* (1 × 10⁸ CFU/200 µL) via alternate-day gavage over a period of six weeks in mice fed with a high-fat, high-carbohydrate (HFC) diet resulted in an increase in microbial richness and diversity, along with a twenty-fold augmentation of *A. muciniphila* in these subjects. Wu et al. [22] demonstrated observed selective modulation following daily gavage of 0.2 mL of live *A. muciniphila* (~ 1.5 × 10¹⁰ CFU/mL) for a duration of 21 weeks, characterized by an increase in *Ruminiclostridium* and *Oscillibacter*, accompanied by a decrease in *Alistipes* and *Lactobacilli*. In a region-specific manner, Morrison et al. [24] reported that heat-inactivated *A. muciniphila* (2 × 10⁸ CFU/mouse) administered daily for 28 weeks resulted in an increase in Bifidobacterium and Enterorhabdus within the colon, as well as Parasutarella in the ileum, while concurrently reducing the populations of Corynebacterium, Desulfovibrionales, Dorea, and Prevotella. Han et al. [23] demonstrate that oral gavage of 0.2 mL of live *A. muciniphila* (1 × 10⁹ CFU/mL) did not significantly influence gut microbiota richness in high-fat diet (HFD) mice. However, continuous quantitative PCR analysis confirmed that A. muciniphila promoted the growth of bacteria that safeguard the gut barrier, without modifying the proportional abundance of the Firmicutes phylum. Oguri et al. [28] demonstrated that a daily oral administration of live ATCC BAA-835 (1 × 10^9 CFU, 200 µL) over four weeks in mice with liver cirrhosis resulted in an improved composition of gut microbiota within a CCl4-induced model of liver cirrhosis. This intervention led to an increase in the abundance of Akkermansia and Muribaculaceae (both negatively associated with liver fibrosis) in the jejunum and ileum, while concurrently decreasing the presence of Streptococcus (which is positively associated with liver fibrosis).

Functionally, *A. muciniphila* influenced SCFA levels. Han et al. [23] observed increased levels of propionic and valeric acids and a restoration of FFAR2 expression in mice fed a high-fat diet, thereby promoting immune homeostasis. Conversely, Morrison et al. [24]found that heat-inactivated *A. muciniphila* reduced valeric and caproic acids. Han et al. [23] reported that there was an enhancement in tryptophan metabolism through the kynurenine pathway, counteracting reductions induced by a high-fat diet. Additionally, Zhuge et al. [31] indicated that twice-weekly gavage of 10⁹ CFU/mouse of live A. muciniphila (ATCC BAA-835) for 20 weeks confers protection against lipid peroxidation and ferroptosis in NASH by modulating the gut microbiota. This process enhances the production of short-chain fatty acids (acetic and propionic acid) via Ruminococcaceae and Roseburia.

#### The impact of *Akkermansia muciniphila* on the metabolism of bile acids

Bile acids (BAs) regulate lipid metabolism and inflammation along the gut-liver axis, and their dysregulation contributes to the progression of Non-Alcoholic Fatty Liver Disease (NAFLD). *A. muciniphila* modulates bile acid metabolism, thereby influencing hepatic cholesterol homeostasis and the clinical outcomes of NAFLD.

Rao et al. [27] Rao et al. demonstrated that alternate-day gavage of live *A. muciniphila* (1 × 10⁸ CFU/200 µL) over a period of six weeks facilitated enhanced bile acid (BA) metabolism and cholesterol absorption within the hepatic system of mice subjected to a high-fat, choline-deficient (HFC) diet, thereby improving the recycling efficiency of the gut-liver axis. Wu et al. [22] observed that daily gavage of 0.2 mL of live *A. muciniphila* (~ 1.5 × 10¹⁰ CFU/mL) over a period of 21 weeks resulted in an increased abundance of Allobaculum. This increase was negatively correlated with specific bile acids (BAs), leading to a reduction in total and secondary BA levels, such as taurochenodeoxycholic acid (TCDCA), lithocholic acid (LCA), and taurodeoxycholic acid (TDCA), in both the caecum and liver. Additionally, hepatic pBAs decreased, while levels of omega-muricholic acid (ω-MCA) and tauro-ω-MCA were restored in mice with MAFLD. Li et al. [25] reported that *A. muciniphila* modified hepatic bile acid composition in mice with non-alcoholic steatohepatitis (NASH), characterized by an increase in primary bile acids such as cholic acid (CA) and tauro-β-muricholic acid (T-β-MCA), as well as a decrease in secondary bile acids including tauro-ω-MCA, TDCA, and tauroursodeoxycholic acid (TUDCA). Han et al. [23] observed a restoration of cholic acid levels in NASH mice fed a high-fat diet, with minimal effects on other bile acids following treatment with live *A. muciniphila* (10⁹ CFU, 200 µL PBS). Furthermore, Wu et al. [30] observed that the administration of live *Akkermansia muciniphila* (5 × 10⁹ CFU/200 µL) over an eight-week period resulted in a reduction of bile acid metabolites, including taurocholic acid, cholic acid, deoxycholic acid, tauroursodeoxycholic acid, and chenodeoxycholic acid. Moreover, this intervention suppressed cholesterol biosynthesis, leading to a decrease in hepatic steatosis. In contrast, Morrison et al. [24] observed that administering heat-inactivated *Akkermansia muciniphila* (2 × 10⁸ CFU per mouse) daily for a duration of 28 weeks did not result in a significant alteration of bile acid elevations induced by a high-fat diet (HFD).

#### Anti-inflammatory effect of *Akkermansia muciniphila*

Inflammatory cytokines, such as tumor necrosis factor-alpha (TNF-α), interleukin-6 (IL-6), IL-1β, IL-1, IL-17, and monocyte chemoattractant protein-1 (MCP-1), contribute to the advancement of NAFLD by fostering insulin resistance, hepatic inflammation, and fibrosis [39–43]. A negative correlation between IL-10 and IL-1β in patients with NAFLD indicates their potential utility as biomarkers for disease progression [40].

Nine studies [19–25, 27–29] Nine studies demonstrated the anti-inflammatory properties of *A. muciniphila.* Que et al. [20] discovered that after a period of ten weeks, administration of pasteurized *A. muciniphila* (1.5 × 10^9 CFU/200 µL) and its outer membrane protein, Amuc_1100, resulted in a reduction of hepatic TNF-α, IL-6, and IL-1β levels in mice fed a high-fat diet (HFD), thereby suppressing inflammatory responses. Wu et al. [19] reported that oral gavage of live *A. muciniphila* (BAA-835, 2 × 10^8 CFU) administered over eight weeks resulted in decreased expression of IL-1β and TNF-α, along with an increase in the anti-inflammatory cytokine IL-10 in mice fed a methionine-choline deficient (MCD) diet, although IL-6 levels were unaffected. Nian et al. [21] observed reductions in serum levels of TNF-α, IL-6, and IL-17 A, accompanied by an increase in IL-10, in mice fed a high-fat diet (HFD) following three weekly administrations of live *A. muciniphila* (0.2 mL, 10⁹ CFU/mL) over an eight-week period. Rao et al. [27] observed a reduction in the gene expression of TNF-α, IL-6, and MCP-1 subsequent to alternate-day administration of live *A. muciniphila* (1 × 10⁸ CFU/200 µL) over a period of six weeks. Concurrently, Wu et al. [22] reported suppressed transcription of these inflammatory genes following daily administration of 0.2 mL of live A. muciniphila (~ 1.5 × 10¹⁰ CFU/mL) for 21 weeks, which contributed to a decrease in intrahepatic inflammation. Han et al. [23] reported that live *A. muciniphila* (10⁹ CFU, 200 µL PBS) reversed the high-fat diet (HFD)-induced upregulation of hepatic inflammatory markers, including Tnf, Ccl2, Ccl3, Ccl7, and Hsp90. Li et al. [25] observed decreased hepatic MCP-1, TNF-α, and IL-6 levels in NASH mice subsequent to the administration of live *A. muciniphila* strain AM06 (10⁹ CFU twice daily for 10 weeks), indicating potential long-term anti-inflammatory effects. Morrison et al. [24] found that heat-inactivated *A. muciniphila* lowered neutrophil chemoattractant KC in the colon and other inflammatory mediators in the ileum and colon.

Hu et al. [29]demonstrated a reduction in the expression of genes associated with inflammation (Tnf, Ccl2) following oral gavage of 0.2 mL live *A. muciniphila* (1 × 10^9 CFU/mL). Conversely, Oguri et al. [28]observed that, after daily gavage of live *A. muciniphila* (ATCC BAA-835, 1 × 10^9 CFU/200 µL PBS with 20% glycerol) for four weeks, levels of Tgfb, Acca, collagen type I-alpha (Col1a1), Tlr2, and Tnfa were significantly lower in the LC-AKK group compared to the LC-NTx2 group. Additionally, Zhuge et al. [31]confirmed that *A. muciniphila* (administered twice weekly at a dose of 10^9 CFU/mouse using the ATCC BAA-835 strain) over a 20-week period activates the AMPK pathway in various tissues, including the liver, ileum, white adipose tissue (WAT), and brown adipose tissue (BAT). The downstream genes SIRT1 and PGC-1α play a crucial role in mediating anti-inflammatory effects through the inhibition of NF-κB.

#### Immune modulatory effect of *Akkermansia muciniphila*

The liver, a critical component of the immune system, modulates inflammation in non-alcoholic fatty liver disease (NAFLD) through the activity of innate and adaptive immune cells, including macrophages, T cells, and natural killer T (NKT) cells. These cells secrete cytokines such as monocyte chemoattractant protein-1 (MCP-1) and tumor necrosis factor-alpha (TNF-α) [44]. Dysregulated immune responses exacerbate NAFLD progression.

Four studies [21, 23, 25, 30] reported that *A. muciniphila* modulates immune responses.

Nian et al. [21]found that administration of three weekly doses of live *A. muciniphila* (0.2 mL, 10⁹ CFU/mL) over an eight-week period resulted in a reduction of T-helper 17 (Th17) cells and an increase in regulatory T (Treg) cells in mice fed a high-fat diet, thereby fostering an anti-inflammatory immune equilibrium. Han et al. [23] demonstrated that oral gavage of 0.2 mL live *A. muciniphila* (1 × 10⁹ CFU/mL) selectively downregulates hepatic toll-like receptor 2 (TLR2) and TLR1 expression, which are altered by a high-fat diet (HFD). This finding suggests an interaction with hepatic macrophages or γδ T cells to suppress microbiota-derived TLR2 signaling, thereby providing protection against non-alcoholic steatohepatitis (NASH). Li et al. [25] observed an increase in hepatic NKT cells, a reduction in CD8 + T cells and macrophages, and an elevation in CXCL16 expression, which facilitates NKT cell accumulation following oral gavage of live A. muciniphila strain AM06 (10⁹ CFU administered twice daily for ten weeks) in NASH mice. These alterations were correlated with a decrease in inflammatory cytokines (MCP-1, TNF-α), suggesting a potential suppression of NASH-associated hepatocellular carcinoma. Additionally, Wu et al.(2025) [30] Additionally, Wu et al. demonstrated that in MAFLD-HCC mouse models, live *A. muciniphila* (5 × 10⁹ CFU/200 µL) administered over eight weeks inhibits LPS influx, downregulates TLR2 and NF-κB, and reduces immunosuppressive M-MDSCs and M2 macrophages, while promoting the presence of Ly6c2-high monocytes, mast cells, and various immune effector cells. *A. muciniphila* diminishes immune-suppressive cytokines (Cxcl1, Ccl2, Cxcl10), thereby fostering a microenvironment conducive to T-cell activity.

**Table 3:** Effects of *Akkermansia muciniphila* supplementation in the management of NAFLD

Reference	Intervention	Control	Dosage and duration	Main outcomes
Hu et al. (2025) [29]	- HFD or CD-HFD + A. muciniphila	- BPS - NC	1 × 10^9^ CFU/mL 4 weeks	- ↓Body weight - ↓The rapidity of body/liver weight gain - ↓Serum TG level - ↓Serum ALT level - ↓ liver impairment and hepatic lipid accumulation - Accelerated liver regeneration, hepatocyte proliferation - ↓The expression levels of genes related to: inflammation (Tnf, Ccl2), fat synthesis (Fasn, Pparg, Acaca, and Scd1), lipid oxidation (Lpl), and fat uptake (Fabp1 and Cd36) in the liver - Maintained gut barrier integrity (↑mucosal thickness - ↑E-cadherin expression- ↓serum LPS levels)
Wu et al. (2025) [30]	1. MAFLD-HCC Model with High-Fat Diet (HFD) and DEN (AKK + HFD + Diethylnitrosamine (DEN) injection) 2. Orthotopic MAFLD-HCC Model with Normal Diet (Akk + Normal diet + RIL-175 cells) 3. Orthotopic MAFLD-HCC Model with MCD Diet (Akk + MCD diet + RIL-175 cells and Hep55.1 C cells mixed with 50% Matrigel) 4. Orthotopic MAFLD-HCC Model with HFHC Diet (Akk + HFHC diet + RIL-175 cells)	- PBS - PBS + IgG2a	5 × 10^9^ CFU/mL 8 weeks (MAFLD-HCC) 2 weeks (DSS)	- ↓ Serum TC and TG + ↓ Bile acid metabolites in the liver, leading to↓ hepatic steatosis - ↑ Intestinal membrane integrity (by increasing tight junction protein expression (ZO-1, Occludin, Claudins 1 and 4) and reducing LPS flux) - suppressed immune-suppressive cytokines (Cxcl1, Ccl2, Cxcl10) - ↓ m-MDSCs and M2 macrophages - ↑ populations of mast cells, plasma cells, dendritic cells, T cells, NK cells, and B cells - suppressed NF-kB pathway and TLR2 expression - ↑effector memory CD4+/CD8 + T cells and exhausted T cell populations (CD4 + PD1+, CD4 + LAG3+, CD8a + PD1+)
Zhuge et al. (2025) [31]	- HFHFD + AKK	-HFHFD -normal control diet	10^9^ CFU/mouse, 20 weeks	- ↓ Weight gain induced by HFHFD - Reversed HFHFD-mediated decreases in AMPK activity in WAT and BAT. - ↑ Insulin sensitivity (↑ IGTT and ↓ fasting insulin) - ↓ Total cholesterol (TC) and ↔ serum TG - ↓Triglyceride abundance and normalized phosphatidylcholine (PC) enrichment - ↑ metabolism of glycerophospholipids (GPs), linoleic acid (LA), α-linolenic acid (α-LA), and arachidonic acid (ARA) based on KEGG enrichment analyses. - Activated hepatic AMPK/SIRT1/PGC-1α axis - ↓ Expression of genes involved in PUFA synthesis (*Fasn*,* Scd1*,* Fads1*,* Elovl4*,* Elovl7*,* Acsl4*) - ↓ ARA-containing and ADA-containing Pes linked to ferroptosis - ↓ Lipid peroxidation markers (MDA, GSH/GSSG ratio improved) - ↓ Pro-inflammatory ω−6 PUFAs (ARA, ADA) - ↓ ALT, ↓ AST - ↓ Hepatic lipid accumulation - ↓ ROS accumulation in the liver and intestine - ↓ NAS score - ↓ Hepatic iron accumulation - ↓ Hepatic ferroptotic markers (↑ Gpx4, ↓ Acsl4) - ↑ Tight junction gene expression - ↑ Mucus layer thickness - ↑ Relative abundance of SCFA producers (Ruminococcaceae, Roseburia) with *A. muc*-derived microbiota - Protective phenotype transferable by FMT from *A. muc*-treated mice
Oguri et al. (2024) [28]	- Akkermansia muciniphila - (RFX)	- Phosphate-buffered saline (PBS) with 10% glycerol (vehicle) - CCl4-induced LC without treatment (LC-NTx) - Olive oil (vehicle) followed by PBS (control-NTx)	- 1 × 10⁹ CFU/day/mouse - 4 weeks	- ↓ Body weight gain - ↓ Liver fibrosis area - ↓ Expression of fibrotic markers (Tgfb, Acca, Col1a1) - ↓blood ammonia (NH3) - ↑ Expression of tight junction genes (Cldn2, Cldn3, Ocln) in small intestine - ↓ Portal endotoxin concentration - ↓ Expression of inflammation markers (Tlr2, Tnfa) - ↓Expression of Tgfb, Acca, and collagen type I-alpha 1 (Col1a1) - The expression of the acetyl-CoA carboxylase alpha gene (Acca) - ↓ Expression of the antimicrobial peptide regenerating islet-derived 3 gamma (Reg3g) in the ileal mucosa - ↑relative abundance of Akkermansia in the cecum and Muribaculaceae in the jejunum and ileum, while reducing Streptococcus in the same regions - No significant change in overall bacterial load was observed, except for an increase in the ileum of male animals
Wu et al. 2024 [19]	- *A. muciniphila* with MCD diet	- SD - MCD diet	2 × 10^8^ CFU 8 weeks	- ↔Body weight - ↓ Level of TG and LDL, ↔ HDL - ↔ ALT levels - ↓ Liver steatosis and ballooning - ↓ Hepatic inflammation - ↓ Expression of proinflammatory cytokines - ↑ Gut barrier function - ↑Relative abundance of proteobacteria - Dominated abundance of *Tannerellaceae*,* Enterobacteriaceae*,* Gammaproteobacteria*, and *Faecalibaculum rodentium*
Qu et al. 2023 [20]	- *A. muciniphila* with HFD	- CD - HFD	1.5 × 10^9^ CFU 10 weeks	- ↓ Body weight - ↓ Serum levels of TC, TG, and LDL-C - ↓ Serum ALT and AST levels - ↓ Lipid droplet vacuole accumulation - ↓ Inflammatory cell infiltration in the liver - ↓ mRNA expression levels of NLRP3, TLR4 and NF-κB - ↓ Concentrations and mRNA levels of TNF α, IL-6, and IL-1β in the liver - ↑ mRNA expression of colonic tight junction proteins - ↓ Concentration of serum LPS
Qu et al. 2023 [20]	- Amuc_1100 with HFD	- CD - HFD	100 µg 10 weeks	- ↓ Body weight - ↓ Serum levels of TC, TG, and LDL-C - ↓ Serum ALT and AST levels - ↓ Lipid droplet vacuole accumulation - ↓ Inflammatory cell infiltration in the liver - ↓ mRNA expression levels of NLRP3, TLR4 and NF-κB - ↓ Concentrations and mRNA levels of TNF α, IL-6, and IL-1β in the liver - ↑ mRNA expression of colonic tight junction proteins - ↓ Concentration of serum LPS - ↓ Abundance of *Coriobacteriaceae_UCG-002* produced by HFD, at the genus level. - ↑ Abundance of *Blautia*,* norank_f__Ruminococcaceae*,* GCA-900,066,575*,* Lachnoclostridium*,* Candidatus_ Saccharimonas and Lachnospiraceae_UCG-006*, at the genus level.
Nian et al. 2023 [21]	- *A. muciniphila* with HFD	- Normal diet - PBS	10^9^ CFU 8 weeks	- ↓Body weight - Improved fasting blood glucose and IR status - ↓ TG and TC levels - ↓ ALT and AST levels - Improved Liver weight and liver index - ↓ Hepatic steatosis and lipid deposition - ↓ Inflammatory cytokine secretion - ↓Intestinal mucosal barrier damage and permeability - ↑ Abundance of *Adlercreutzia* - ↓ Abundance of *Romboutsia*,* Lactobacillus*, and *Parabacteroides.* - Induced transformation of T cells to Treg
Wu et al. 2023 [22]	- *A. muciniphila* with HFD	- LFD - HFD	~ 1.5 × 10^9^ CFU 21weeks	- Inhibited HFD-induced weight gain - Improved adipocyte metabolism and related adipokines - Improved glucose tolerance - ↓ serum ALT and AST - Improved liver volume due to decreased lipid deposition - ↓ Histological NAS score - ↓ Concentration of intrahepatic TGs - ↓ Intrahepatic inflammation - ↑ Intestinal barrier function - ↓ Endotoxin translocation - ↑ Ruminiclostridium, *Osclibacter*,* Allobaculum*,* Anaeroplasma*, and *Rikenella* - ↓ *Alistipes*,* Lactobacilli*,* Tyzzerella*,* Butyricimonas*, and *Blautia* - Inhibited hepatic bile acid synthesis - Altered structure of bile acid profiles - ↓Primary and secondary BAs
Han et al. 2023 [23]	- *A. muciniphila* with HFD	- Normal chow diet - HFD	10 ^9^ CFU 20 weeks	- ↓ Body weight without effect on food intake (after 16 weeks, in the HFD group) - ↓ Serum GLU and TC levels (At the 18th week, in the HFD group) - ↑GLP-1 level - ↓ ALT and AST levels - ↓ Liver steatosis and inflammatory cell infiltration, and NAS - ↓ Lipid accumulation in the liver - Slightly improved hepatic fibrosis - ↓ Expression of hepatic inflammatory markers - Improved intestinal barrier function by upregulated expression of tight junction proteins, increased mucus production, and reduced LPS - No regulatory effect on commensal diversity and richness - Regulatory effect on the composition of the gut microbiota by upregulation of gene expression of antibacterial peptides - Flourishing of intestinal *A. muciniphila* - Controlled γδT accumulation and macrophage polarization
Morrison et al. 2022 [24]	- Pasteurized and lyophilized *A. muciniphila* with HFD	- Standard rodent maintenance diet - NASH-inducing HFD	2 × 10^8^ CFU 28 weeks	- ↔ Body weight, adiposity and food intake - ↓ Hypertrophy in the mesenteric WAT - ↔ plasma lipids, blood glucose, or plasma insulin - ↔ Hepatic inflammation, steatosis and fibrosis - Minor Effects on Inflammation in Ileum and Colon - ↑ Gut integrity and gut barrier function - ↔ Bile acids In the colon mucosa: - ↑ Abundance of *Enterorhabdus* and *Bifidobacterium* - ↓ Abundance of *Corynebacterium*,* Desulfovibrionales*, and *Dorea* In the ileum mucosa: - ↑Abundance of *Bifidobacterium* and *Parasutarella* - ↓ Abundance of *Prevotella*
Li et al. 2022 [25]	- *A. muciniphila* (AM06) * with HFD	- Normal diet - PBS	1.0 × 10^9^ CFU 16 weeks	- ↔ Body weight - ↓Fasting blood glucose level - ↔Insulin - ↑GLP-1 level - ↔ TG - ↑ALT ↔ AST (end of 10 weeks) ↔ALT ↓AST (end of 20 weeks) - ↓ Score of steatosis, ballooning, and NAS - ↓Fatty degeneration in the liver - ↓Hepatic steatosis, inflammation, and necrosis - ↓ IL-6 levels - ↓Inflammatory factors of MCP-1 and TNF-α expression in the liver - ↑ Primary bile acids and↓ Secondary bile acids - ↑NKT cell accumulation in the liver - ↑Hepatic CXCL16 expression - ↓Macrophage accumulation in the liver - ↓CD8 + T cells - ↔ Monocytes
Juárez-Fernández et al. 2021 [26]	- *A. muciniphila* with HFD - *A. muciniphila* with control diet	- CD - HFD	2 × 10^8^ CFU 3 weeks	- ↓ White adipose tissue - ↓ Liver TG concentration - ↑ Abundance of the *Akkermansia* genus - ↑ Abundance of the *Verrucomicrobia* phylum
Rao et al. 2021 [27]	- *A. muciniphila* with HFC - *A. muciniphila* with HFC and ABx	- HFC - HFC + Abx	1 × 10^8^ CFU 6 weeks	- ↓ Body weight - ↓ Plasma and hepatic cholesterol levels - ↓ Plasma levels of ALT, AST, and ALP - ↓ Hepatic steatosis - Suppressed expression levels of genes related to steatosis and inflammation - Maintained gut barrier integrity - ↑ Richness and diversity of gut microbiota - Improved microbial community structure - ↓ Bile acid metabolism dysfunction in the gut-liver axis

↑ increased, ↓ decreased, ↔ not changed, *Abx* antibiotics mixture, *AST* aspartate amino transferase, *ALP* Alkaline phosphatase, *ALT* alanine amino transferase, *BAs* bile acids, *CFU* colony-forming unit, *CD* control diet, *GLU* glucose, *γδT* a type of T cells, *GLP-1* Glucagon like peptide, *HDL* high density lipoprotein, *HFC* high-fat and high-cholesterol, *HFD* High fat diet, *IL-1β* Interleukin-1beta, *IL-6* Interleukin 6, *LDL* low density lipoprotein, *LFD* low fat diet, *LPS* Lipopolysaccharides, *MAFLD* metabolic-associated fatty liver disease, *MCD* methionine-choline-deficient, *MCP-1* monocyte chemoattractant protein, *NAFLD* non-alcoholic fatty liver disease, *NAS* NAFLD activity score, *NASH* non-alcoholic steatohepatitis, *NF-κB* nuclear factor κB, *NLRP3* nodular receptor protein 3, *PBS* phosphate-buffered saline, *SD* standard diet, *SPF* specific pathogen free, *TC* total cholesterol, *TG* triglycerides, *TLR4* Toll-like receptor 4, *TNF-α* Tumor Necrosis Factor Alpha, *WAT* white adipose tissue

*Results of this study initially showed that AKK obtained from human breast milk (AM06) is more effective than AKK derived from feces (AM02) in preventing NAFLD. Thus, *A. muciniphila *produced from breast milk was employed in all subsequent investigations [25]

## Discussion

This systematic review comprehensively examines the effects and mechanisms of *A. muciniphila* on NAFLD mouse models. A total of 13 studies were selected for inclusion. Given the generally low to moderate quality of the included studies and the potential heterogeneity among them, the results should be interpreted with caution. Based on the findings, *A. muciniphila* demonstrates potential as a next-generation probiotic for the management of NAFLD. It offers beneficial effects, including reductions in body weight, hepatic steatosis, and serum lipid levels (such as triglycerides and cholesterol), while also improving insulin sensitivity and liver enzyme levels (ALT, AST) in models fed a high-fat diet (HFD). The microorganism enhances gut barrier integrity by upregulating tight junction proteins (e.g., ZO-1, Occludin) and decreasing lipopolysaccharide (LPS) translocation, thereby alleviating hepatic inflammation through the gut-liver axis [19–23, 27]. Additionally, A. muciniphila modulated bile acid metabolism by increasing primary bile acids and decreasing secondary bile acids. Furthermore, it altered the composition of gut microbiota, enriching beneficial taxa such as Lachnospiraceae and Ruminococcaceae [20, 22, 27]. However, heat-inactivated A. muciniphila demonstrated limited effectiveness, especially for steatosis [24]., It is suggested that live bacteria possess greater efficacy.

*A. muciniphila*,* a beneficial bacterium*,* is essential for maintaining the integrity and function of intestinal mucus. It decomposes mucins to extract carbon*,* nitrogen*,* and energy*,* resulting in the production of oligosaccharides and short-chain fatty acids (SCFAs)*,* which support its metabolic processes as well as those of the host. Additionally*,* it alleviates inflammatory responses and reduces intestinal permeability by producing SCFAs*,* activating G protein-coupled receptors (GPRs)*,* and inhibiting histone deacetylases (HDACs)* [45]. Fecal microbiota transfer from A. muc-treated mice or colonization in germ-free mice reverses diet-induced hepatic SCFA reductions, activating the AMPK/SIRT1/PGC-1α pathway, reducing ROS and ferroptosis markers. A. muc uniquely elevates Acss1 levels, thereby enhancing AMPK activity for an anti-ferroptotic effect effect [31].

Mechanistically, *A. muciniphila* suppresses lipogenic genes (e.g., FAS, ACC), elevates L-aspartate levels within the gut-liver axis, subsequently activates the LKB1-AMPK pathway, thereby enhancing lipid oxidation, reducing cell death induced by oxidative stress, and promoting bile acid regulation metabolism [27]. It elevates glucagon-like peptide-1 (GLP-1) levels, counteracting the upregulation of dipeptidyl peptidase IV (DPPIV) induced by a high-fat diet (HFD), and regulates bile acid signaling through the farnesoid X receptor (FXR) and fibroblast growth factor 15 (FGF15), thereby decreasing hepatic cholesterol synthesis [22, 23]. Its anti-inflammatory effects involve downregulating TNF-α, IL-6, and MCP-1, while upregulating IL-10. Additionally, its immunomodulatory properties include balancing Th17/Treg cells and suppressing TLR2 signaling [23, 25]. It downregulates hepatic TLR2 signaling, thereby reducing γδT17 cell levels and IL–17–mediated M1 macrophage polarization. Consequently, this inhibition impedes the progression of NASH [23]. 

This study possesses several limitations. Firstly, heterogeneity in study design—including variations in bacterial strains, dosages, administration routes, and dietary models—impedes direct comparison and may contribute to inconsistent outcomes. Secondly, dependence on rodent models restricts the applicability of findings to humans, owing to differences in metabolism and microbiota that may affect responses. Nonetheless, an exploratory human study demonstrated that *A. muciniphila* diminished markers of liver dysfunction in obese individuals, thereby indicating translational potential [46]. Thirdly, this review encompassed multiple aspects of NAFLD; it did not address the anticancer effects of A. muciniphila or the underlying mechanisms.

A current study investigates the therapeutic potential of lyophilized fecal microbiota capsules (LFMCs) sourced from healthy vegan donors, which include a combination of next-generation beneficial bacteria, such as pasteurized A. muciniphila, in individuals with steatohepatitis [47]. The isolated effect of *A. muciniphila* in patients with NAFLD remains unexamined and warrants further investigation. Mechanistic studies exploring *A. muciniphila*’s interactions with the gut microbiota and bile acid pathways, as well as comparative analyses with traditional probiotics, are essential for elucidating its potential therapeutic role.

## Conclusion

*A. muciniphila* is emerging as a prospective therapeutic target for NAFLD, with potential to enhance glucose and lipid metabolism, hepatic function, gut barrier integrity, and immune-inflammatory responses in animal models. Its mechanisms encompass the modulation of the gut-liver axis, bile acid metabolism, and microbial community composition. Nonetheless, the absence of clinical data highlights the necessity for comprehensive trials to establish optimal formulations, dosages, and safety profiles.

## Data Availability

The authors confirm that the data supporting the findings of this study are available within the article and its supplementary materials.

## Abbreviations

A. muciniphila: Akkermansia muciniphila
ACC: Acetyl-CoA Carboxylase
Acss1: Acetyl-CoA Synthetase 1
AKK: Akkermansia muciniphila
ALP: Alkaline Phosphatase
ALT: Alanine Aminotransferase
AMPK: AMP-Activated Protein Kinase
AST: Aspartate Aminotransferase
BA: Bile Acid
BAT: Brown Adipose Tissue
CA: Cholic Acid
CCl_4_: Carbon Tetrachloride
CCL2: Chemokine (C-C motif) Ligand 2
CCL3: Chemokine (C-C motif) Ligand 3
CCL7: Chemokine (C-C motif) Ligand 7
CDCA: Chenodeoxycholic Acid
CFU: Colony-Forming Unit
Cldn: Claudin
Col1a1: Collagen Type I Alpha 1
CXCL1: C-X-C Motif Chemokine Ligand 1
CXCL10: C-X-C Motif Chemokine Ligand 10
CXCL16: C-X-C Motif Chemokine Ligand 16
DCA: Deoxycholic Acid
DPPIV: Dipeptidyl Peptidase IV
FAS: Fatty Acid Synthase
FDA: Food and Drug Administration
FD4: FITC-Dextran 4
kDaFFAR2: Free Fatty Acid Receptor 2
FGF15: Fibroblast Growth Factor 15
FXR: Farnesoid X Receptor
GLP-1: Glucagon-Like Peptide-1
GPR: G Protein-Coupled Receptor
GTT: Glucose Tolerance Test
HCC: Hepatocellular Carcinoma
HDAC: Histone Deacetylase
HFC: High-Fat, High-Carbohydrate (diet)
HFHFD: High-Fat, High-Cholesterol, High-Fructose Diet
HFD: High-Fat Diet
HFHC: High-Fat, High-Cholesterol (diet)
HIV: Human Immunodeficiency Virus
HSP90: Heat Shock Protein 90
IGTT: Insulin and Glucose Tolerance Test
IL-1: Interleukin-1
IL-1β: Interleukin-1 Beta
IL-6: Interleukin-6
IL-10: Interleukin-10
IL-17: Interleukin-17
IL-17A: Interleukin-17A
LBP: Lipopolysaccharide-Binding Protein
LC-AKK: Liver Cirrhosis + Akkermansia muciniphila Treatment
LC-NTx2: Liver Cirrhosis + Non-Treatment Control
LCA: Lithocholic Acid
LDL-C: Low-Density Lipoprotein Cholesterol
LFMC: Lyophilized Fecal Microbiota Capsule
LKB1: Liver Kinase B1
LPS: Lipopolysaccharide
MAFLD: Metabolic Dysfunction-Associated Fatty Liver Disease
MCD: Methionine-Choline-Deficient (diet)
MCP-1: Monocyte Chemoattractant Protein-1
MDSC: Myeloid-Derived Suppressor Cell
M-MDSC: Monocytic Myeloid-Derived Suppressor Cell
Muc2: Mucin 2
NAFLD: Non-Alcoholic Fatty Liver Disease
NAS: NAFLD Activity Score
NASH: Non-Alcoholic Steatohepatitis
NF-κB: Nuclear Factor Kappa B
NH_3_: Ammonia
NKT: Natural Killer T (cell)
Ocln: Occludin
PGC-1α: Peroxisome Proliferator-Activated Receptor Gamma Coactivator 1-Alpha
PRO-C4: Procollagen Type IV C-terminal Propeptide
ROS: Reactive Oxygen Species
SCFA: Short-Chain Fatty Acid
SIRT1: Sirtuin 1
STZ: Streptozotocin
T-β-MCA: Tauro-β-Muricholic Acid
TC: Total Cholesterol
TCDCA: Taurochenodeoxycholic Acid
TDCA: Taurodeoxycholic Acid
TG: Triglyceride
TGFB: Transforming Growth Factor Beta
Th17: T-Helper 17 (cell)
Tjp1: Tight Junction Protein 1
Tjp3: Tight Junction Protein 3
TLR1: Toll-Like Receptor 1
TLR2: Toll-Like Receptor 2
TNF-α: Tumor Necrosis Factor Alpha
Treg: Regulatory T (cell)
TUDCA: Tauroursodeoxycholic Acid
WAT: White Adipose Tissue
ZO-1: Zonula Occludens-1
ZO-2: Zonula Occludens-2
ω-MCA: Omega-Muricholic Acid
